# Tulane Virus Persistence and Microbial Stability in 3D Food Ink under Various Storage Conditions: A Pre- and Post-Printing Analysis

**DOI:** 10.1007/s12560-024-09597-0

**Published:** 2024-05-06

**Authors:** Allyson N. Hamilton, Kristen E. Gibson

**Affiliations:** Department of Food Science, Center for Food Safety, University of Arkansas System Division of Agriculture, 1371 West Altheimer Dr, Fayetteville, AR 72704 USA

**Keywords:** 3D printed food safety, Foodini, Capsule, Protein cookie food ink

## Abstract

3D food printers facilitate novel customization of the physicochemical properties of food. This study aimed to investigate the impact of storage conditions on the inactivation of the human norovirus surrogate, Tulane virus (TuV), within 3D printed foods. TuV-inoculated protein cookie food ink (∽ 4 log PFU/g) was distributed into 18 3D food printer capsules (50 g each); half immediately underwent extrusion. Storage of the capsules and printed food products at 20 °C (0, 6, 12, and 24 h), 4 °C (0, 1, 3, and 5d), and − 18 °C (0, 1, 3, and 5d) was completed before analysis for TuV via plaque assays in addition to aerobic plate count, yeast and mold counts, and pH and water activity (a_w_) measurements. A significant 3-way interaction effect was observed between time, temperature, and storage method (capsule/print) (*p* = 0.006). Significant findings include: (1) A greater reduction in virions was observed in capsules after 24 h at 20 °C and (2) a substantial reduction in virions at 4 °C from day 0 to day 1 was observed, independent of storage method. Microbial indicators remained steady across temperatures, with storage temperature significantly impacting pH and a_w_. A significant two-way interaction effect (*p* = 0.006) was found between microorganism type (yeast/aerobic counts) and temperature. This research seeks to provide insights for the food industry and regulatory bodies in crafting guidelines for the safe storage and handling of 3D printed foods and inks.

## Introduction

The global landscape of 3D food printing (3DFP), valued at an estimated USD$15 billion in 2023 and projected to escalate to USD$34.5 billion by 2028 (Markets and Markets, 2023), represents the forefront of culinary evolution, promising a paradigm shift in how we perceive and engage with food. This cutting-edge technology allows for the precise customization of food, covering aspects such as texture, color, shape, and various physiochemical attributes (Sun et al., 2015). Within this burgeoning field, a myriad of food materials has already undergone 3DFP processes. From the layers of printed cheese and cookies to surimi, chocolate, turkey, and even cell-cultured meat, the possibilities are limitless, signifying an era of diverse culinary exploration (Dick et al., 2020; Handral et al., 2020; Lanaro et al., 2017; Le Tohic et al., 2018; Mantihal et al., 2019; Ross et al., 2021; Severini et al., 2018; Vukušić Pavičić et al., 2021; Wang et al., 2018; Wilson et al., 2020; Yang et al., 2018).

Nevertheless, as with any innovation, the assimilation of 3DFP demands the evaluation of potential hazards to ensure the safety and wholesomeness of the resulting food products. The application of 3DFP introduces distinct challenges stemming from increased handling requirements, thus presenting opportunities for contamination within both the food ink and within its corresponding capsules (Hamilton & Gibson, 2022, 2023a, b). The procedure for producing 3D printed foods encompasses the conventional protocols of food handling but also extends to the loading of food ink capsules and the subsequent post-printing handling, potentially exacerbating the risk of cross-contamination between the capsule, the food ink, and the food preparation environment.

Annually, major causative agents of foodborne illnesses contribute to an estimated 9.4 million cases and over 1,300 fatalities in the United States, alongside a staggering 600 million illnesses and over 418,000 deaths globally (Havelaar et al., 2015; Scallan et al., 2011). The predominant viral pathogen of concern is human norovirus (HuNoV). HuNoV not only poses a threat in the context of 3D printed foods but also stands as a significant contributor to foodborne illnesses both in the United States and on a global scale. HuNoV accounts for 58% (5.4 million) of reported foodborne illnesses, 149 fatalities in the United States, and an imposing 125 million illnesses and 35,000 deaths worldwide (Havelaar et al., 2015; Scallan et al., 2011). The economic repercussions of HuNoV in the United States are noteworthy, constituting a substantial $2.9 billion out of the total $51.05 billion attributed to all pathogens associated with foodborne illnesses, establishing it as the primary viral contributor to this economic burden (Scharff, 2012).

HuNoV is primarily transmitted through oral ingestion, leading to replication within the small intestine. This process results in lesions across the intestinal mucosa and subsequent viral shedding via feces (Tian et al., 2013). The critical role of histo-blood group antigens (HBGAs) as receptors facilitating HuNoV attachment and entry into host cells was elucidated by Tan and Jiang (2005). HuNoVs demonstrate strain-specific recognition of HBGAs, categorized into three major families: secretor, Lewis, and ABO (Tan & Jiang, 2010). Despite the significant public health risks posed by HuNoVs, research into their behavior and control is hampered by the challenges associated with culturing these viruses and the absence of effective small animal models. Recent advancements in three-dimensional cell culture technologies, particularly non-transformed stem cell-derived human intestinal enteroids, have shown promise. These enteroids mimic the gastrointestinal tract’s complexity and cellular diversity, offering new pathways for HuNoV research (Chan et al., 2019; Costantini et al., 2018; Ettayebi et al., 2016).

The material properties of a_w_ and pH may influence the persistence of viruses, as evidenced by prior studies (Tian et al., 2013; Trudeau et al., 2017). Foods and food ingredients with low a_w_ are either intrinsically low in moisture or deliberately dehydrated from initially high- a_w_ sources (Beuchat et al., 2013). The incorporation of significant quantities of salt or sugar can also be construed as an engineered drying process, resulting in a diminished water availability that impedes microbial growth (Beuchat et al., 2013). It is noteworthy that the minimum a_w_ conducive to the growth of specific microorganisms, such as molds and yeasts, is established at 0.60 (Beuchat et al., 2013). Food products characterized by an a_w_ < 0.85 fall into the category of low-a_w_, encompassing a diverse range including cereals, chocolate, dried produce, animal feed, spices and condiments, honey, hydrolyzed protein powder, pasta, peanut butter, and seeds (Beuchat et al., 2013). Despite the evident benefits of low-a_w_ foods in controlling bacterial foodborne pathogens, there remains a scarcity of research on their influence on viral foodborne pathogens (Franco-Vega et al., 2020; Roos, 2020).

Given the difficulties in studying HuNoVs directly, surrogate viruses have been employed to understand HuNoV persistence and resistance. Initially, surrogates included poliovirus, hepatitis A virus (HAV), and bacteriophages as stand-ins for HuNoVs inactivation studies (Baert et al., 2009). Then, investigations turned to animal caliciviruses, such as feline calicivirus (FCV) and murine norovirus (MNV), due to their cultivability (Kreutz et al., 1994; Wobus et al., 2004). However, these surrogates lack the HBGA receptors akin to the predominant circulating HuNoV strains (e.g., GII.4) and do not induce gastrointestinal disease, marking a significant departure from HuNoV pathogenesis. Interestingly, while MNV shares genetic similarities with HuNoVs, it utilizes sialic acid receptor for entry and infecting macrophages and dendritic cells rather than mimicking HuNoV’s gastrointestinal targets (Taube et al., 2009; Wobus et al., 2004). On another front, Tulane virus (TuV) offers a closer surrogate model by replicating in vitro in rhesus monkey kidney (LLC-MK2) cells and recognizing type-B HBGAs for infection, aligning more closely with HuNoV’s enteric nature (Farkas et al., 2008, 2010).

The primary aim of this study was to investigate the impacts of varying storage conditions, including temperature, time, and storage method (pre- vs. post-printing), on persistence of TuV, a HuNoV surrogate. The secondary aim is to evaluate food ink quality using four parameters: yeast and mold counts, aerobic counts, pH, and water activity (a_w_). To achieve these aims, 4 log PFU/g of TuV was incorporated into a protein cookie food ink for 3DFP, and analyses were conducted to assess its safety and quality, providing essential insights for development of regulatory guidance on 3D printed food inks and printed products.

## Materials and Methods

### Tulane Virus Propagation

Tulane virus (provided by Dr. Jason Jiang, Cincinnati Children’s Hospital Medical Center, Cincinnati, OH) was propagated in LLC-MK2 cells (ATCC CCL-7) (American Type Culture Collection, Manassas, VA) as previously described by Arthur and Gibson (2015). Briefly, LLC-MK2 cells were cultivated and maintained in Medium 199 with Earle’s Balanced Salt Solution (EBSS) (Hyclone, Logan, UT) supplemented with 1% Penicillin/Streptomycin (Hyclone), 1% Amphotericin B (Corning, Mediatech Inc., Manassas, VA), and 10% Fetal Bovine Serum (FBS) (Hyclone).

For virus cultivation, a TuV stock, prepared at a multiplicity of infection (MOI) of 0.1 in 6 mL of Opti-MEM (Gibco Life Technologies, Grand Island, NY), was introduced into monolayers of LLC-MK2 cells that had reached 90% confluency. These cells were then incubated with continuous rocking at 5% CO2 and 37 °C for one hour. Following this, an additional 20 mL of Opti-MEM supplemented with 2% FBS was introduced, and the cells were maintained in a T150 flask under the same conditions until a complete cytopathic effect was evident, typically around 60 h post-inoculation. After this incubation period, the flask was frozen at -80 °C overnight. TuV was then extracted through a series of three freeze-thaw cycles, followed by centrifugation at 3,000 × g for 15 min at 4 °C. The resulting supernatant was then filtered using a Steriflip® 0.45 μm filter (Millipore, Burlington, MA) and aliquoted into 1 mL cryovials, which were subsequently stored at -80 °C.

### Quantification of TuV

To determine the concentration of TuV (Arthur & Gibson, 2015), LLC-MK2 cells, at a density of 4 × 10^5^ cells per well, were seeded into six-well tissue culture-treated plates and incubated for 24 h at 37 °C in a 5% CO_2_ atmosphere. Following this, the media was discarded and each well received 500 µL of TuV stock, serially diluted from 10^− 1^ to 10^− 6^ in Opti-MEM enriched with 2% FBS. The plates were then incubated for one hour at 37 °C under 5% CO_2_ with gentle rocking. Simultaneously, an overlay mixture was prepared by combining an equal volume of 3% low-melting NuSieve™ GTG™ Agarose (Lonza, Walkersville, MD) in its liquefied state with Opti-MEM also supplemented with 2% FBS. Post-incubation, the inoculum was aspirated from the wells, and each well was overlaid with 2 mL of the overlay mixture. This overlay was allowed to solidify for 15 min, after which the plates were incubated at 37 °C and 5% CO_2_ until plaque formation, typically within 96–120 h. For plaque visualization, a 3% solution of neutral red stock (Sigma-Aldrich, St Louis, USA) in 1X PBS was prepared. Two milliliters of this staining solution were added to each well, followed by an incubation period of 1–3 h at 37 °C under 5% CO_2_. Post-incubation, the stain was removed, and plaque forming units (PFUs) were enumerated. The resulting concentration of the final TuV stock was determined to be approximately 10^6^ PFU/mL.

### Inoculated Food Ink Preparation

To create the protein cookie food ink, 560 g of room temperature unsalted butter (Member’s Mark Unsalted Sweet Cream Butter; Sam’s West, Inc, Midwest City, OK) was placed into a KitchenAid 6 Quart Bowl-Lift Stand Mixer (KitchenAid, Benton Harbor, MI). To this, 500 g of granulated sugar (Great Value, Bentonville, AR), 300 mL of deionized water, 310 g of protein powder (Sports Research Collagen Powder Supplement; Sports Research, San Pedro, CA), and 300 g of bleached and enriched all-purpose flour (Great Value) were added. The mixture of these components was then thoroughly combined in the stand mixer for a duration of 15 min. Following this initial mixing phase, 4 mL of TuV stock (10^6^ PFU/mL), was incorporated into the food ink. The mixture was then subjected to an additional 15 min of mixing to ensure even distribution of the virus stock within the food ink.

### Preparation of the Food Ink Capsules

The Foodini 3D Food Printer, has been extensively described in previous works by Hamilton and Gibson (2022, 2023a, b). In summary, the Foodini is designed to be a versatile, countertop 3D food printer suitable for both domestic and professional culinary environments. The primary food-contact surface is a cylindrical stainless steel food ink capsule, with a length dimension of 11 cm and diameter of 4 cm. The term ‘food ink’ in this context refers to the edible material extruded from this capsule, specifically, the protein cookie food ink utilized in the current manuscript. As per the guidelines stipulated in the Foodini user manual, it is recommended to limit the volume of food ink in each capsule to a maximum of 100 mL (https://static.naturalmachines.com/images/Natural-Machines-Foodini-Brochure.pdf).

The sanitization protocol for the stainless-steel capsules was conducted in accordance with the methodology established by Hamilton and Gibson (2022). This protocol entailed an initial cleaning of the capsules using Dawn Ultra dish soap (Proctor and Gamble, Cincinnati, OH), followed by a rinse with deionized water, and a subsequent sanitization using a 70% ethanol solution. After the cleaning procedure, the capsules were left to air dry. To achieve complete sterilization, the capsules were then autoclaved at a temperature of 121 °C for a duration of 15 min, sealed within sterilization pouches.

### Loading, Storing, and Sampling of the Food Ink Capsules and Printed Food Ink

To load the 3DFP capsules, 50 ± 1 g of the protein cookie food ink were added into each capsule, followed by the secure placement of the capsule caps. Given the absence of established storage guidelines and anticipating the practical needs of high-volume printing operations in restaurants, half of these prepared capsules were stored horizontally inside airtight food storage containers. This horizontal orientation was selected deliberately to prevent any leakage of food ink from the capsule tip. This pre-filling and storage strategy is designed to streamline the printing process, offering significant time savings. The remaining half of the capsules were extruded onto flattened stomacher bags (Filtra-bag® with tear-off top, 7 × 12 in., VWR, Radnor, PA). This extrusion was achieved by pressing the capsule cap towards the base of the capsule, and the resultant printed food products were then stored in airtight containers, ensuring the final weight of these products was consistently maintained at 50 ± 1 g.

**Fig. 1:**
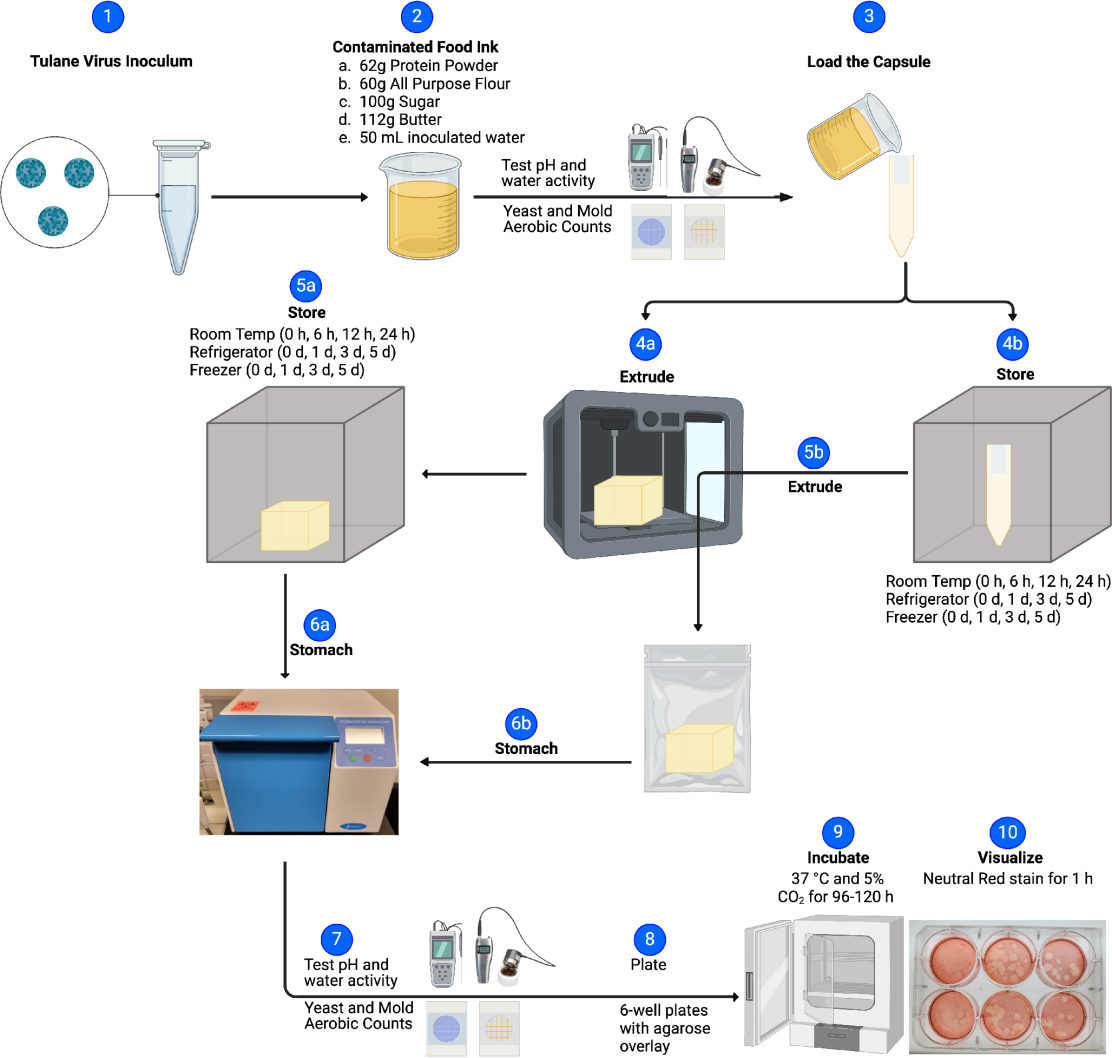
Schematic of experimental flow. Created in Biorender.com

The storage conditions for both the filled capsules and the extruded food ink products were for durations of 0, 1, 3, and 5 days at temperatures of 4 °C and − 18 °C, or for periods of 0, 6, 12, and 24 h at 20 °C. These times and temperatures were selected as they represent storage at room temperature, in a refrigerator, and in a freezer for intervals food is commonly stored by consumers (Terpstra et al., 2005). For the purpose of enumeration, at the designated time points, the contents of the capsules were extruded onto flattened stomacher bags (Filtra-bag® with tear-off top, 7 × 12 in., VWR, Radnor, PA) by pressing the capsule cap down to the capsule base. To each of these bags, 50 mL of 1X PBS was added, encompassing both the extruded capsule contents and the printed food products. The contents of the bags were then homogenized using a stomacher for 1 min at 230 revolutions per minute. The homogenate was subsequently serially diluted in Opti-MEM supplemented with 2% FBS and analyzed using the plaque assay methodology detailed in the section “Quantification of TuV” (Fig. 1). The samples from timepoint 0 were considered as the baseline. Serially diluted TuV stock was considered as positive control, and for negative control, the study included uninoculated food ink, 1X PBS, and Opti-MEM enriched with 2% FBS.

### Quality Indicator Analysis

To assess the quality and potential spoilage of the food ink, a_w_ and pH were measured at the initial and final time points of the experiments. Water activity was determined using the AquaLab TDL (LABCELL Ltd., United Kingdom), while pH measurements were conducted with the Fisherbrand™ accumet™ AB200 Benchtop pH meter (Fisher Scientific, Hampton, NH). Furthermore, to evaluate microbial proliferation, aerobic bacterial populations were quantified using 3 M™ Petrifilm® Aerobic Count Plates (3 M, St. Paul, MN). Similarly, the presence and growth of yeasts were monitored using 3 M™ Petrifilm® Yeast and Mold Count Plates (3 M). These microbial counts were performed at each designated time point (Fig. 1). This comprehensive approach ensured a thorough assessment of food quality and safety over the duration of the experiment.

### Statistical Analysis

Two experimental trials with three samples each were performed for each time, temperature, and storage method combination (*n* = 6 per combination). Statistical analysis was computed to determine if storage time, storage temperature, or storage method were significant predictors of TuV level in the final protein cookie product. Further statistical analysis was completed for quality indicator microorganisms to determine if storage time and storage temperature were significant predictors of quality indicator microorganism levels in the final protein cookie product.

Additional statistical analyses were computed to determine if storage time or storage temperature were significant predictors of pH or a_w_. Data were analyzed in R Studio (R Studio Team, 2022) using a linear model (ANOVA) for TuV and quality indicator microorganisms. These residual analyses met the assumptions of normality and homoscedasticity. Water activity and pH did not meet the assumption of normality, so the data were then analyzed using a generalized linear model (GLM) with Poisson error distributions. The respective residual deviances were less than the respective residual degrees of freedom, which indicated that the GLMs properly estimated the variance of the pH and a_w_ data. The modeled means and their associated standard errors were calculated using estimated marginal means (when results were significant at an α-level of 0.05), and statistical differences between treatments were determined using multiple comparisons and visualized using compact letter display.

The data were analyzed in R Studio using the base (R Core Team, 2022), ggplot2 (Wickham & Sievert, 2016), emmeans (Length et al., 2021), tidyverse (Wickham et al., 2019), ggpubr (Kassambara, 2020), gdata (Warnes et al., 2022), rstatix (Kassambara, 2021), lme4 (Bates et al., 2015), lmertest (Kuznetsova et al., 2017), multcomp (Hothorn et al., 2008), and multcompView (Graves et al., 2019) packages.

## Results

**Fig. 2:**
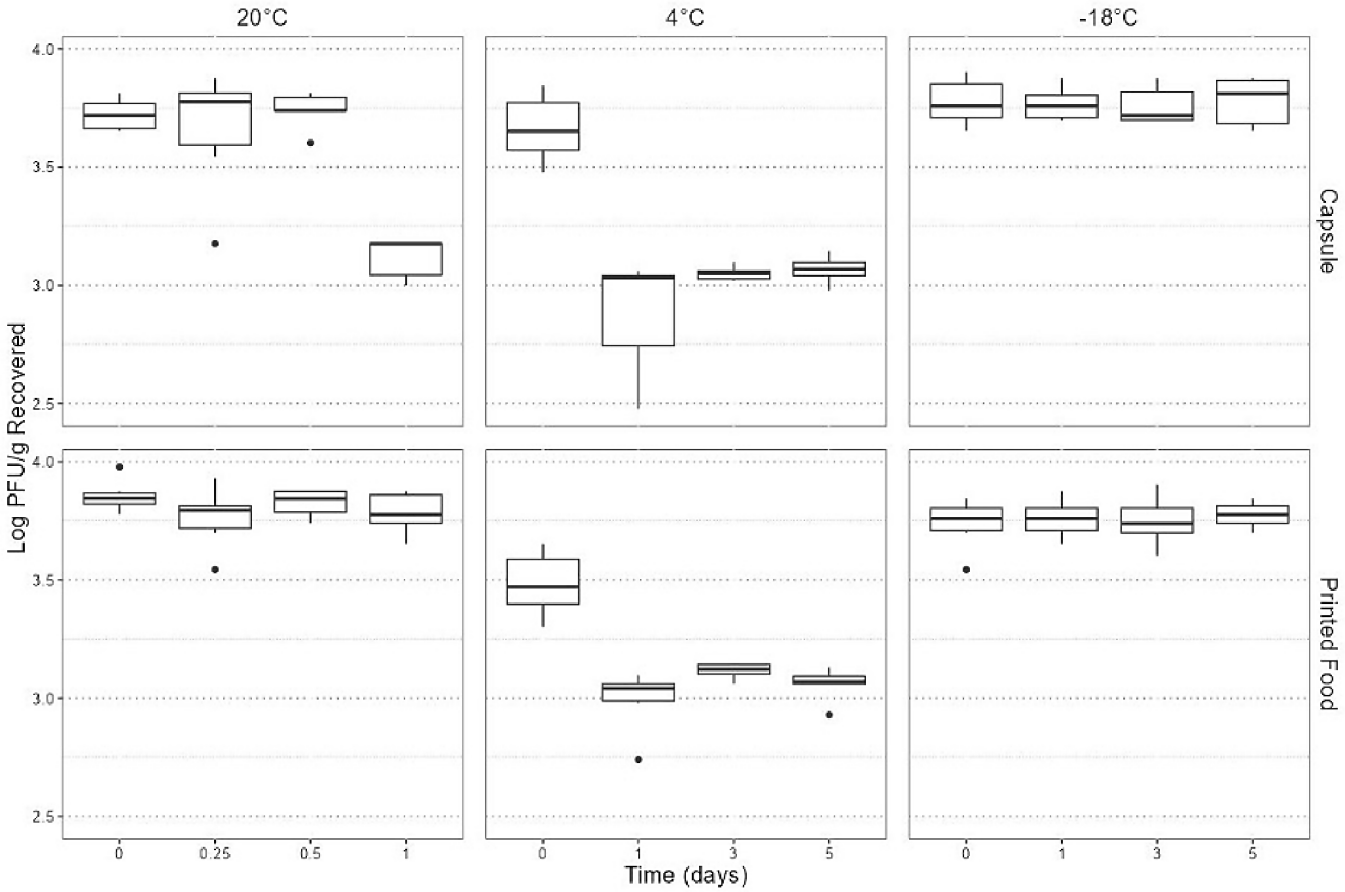
Raw data for Tulane virus based on time, temperature, and storage method parameters. In the box plots, the boundary of the box closest to 0 indicates the 25th percentile, the middle line within the box marks the median, and the boundary of the box farthest from zero indicates the 75th percentile. Whiskers above and below the box indicate 1.5 times the interquartile range (the distance between the 25th and 75th percentiles) but are limited to reaching actual data points. Points outside of the boxplots are outliers, that is, values that are outside the range of the whiskers

**Fig. 3:**
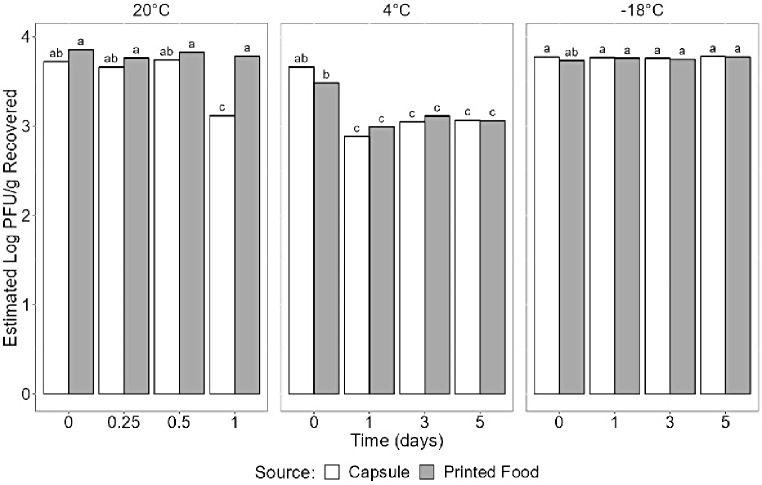
Statistical analysis using ANOVA for Tulane virus based on time, temperature, and storage method parameters. Compact letter format is used to designate statistical differences between treatments at *p* = 0.05

Figure 2 displays the comprehensive visualization of raw data pertaining to TuV as logarithmic PFU/g values recovered from the protein cookie product across varying storage times, temperatures, and recovery locations (capsule or printed food). Employing a linear model to analyze the data revealed a noteworthy three-way interaction effect among time, temperature, and storage method (capsule/print) (*p* = 0.006). This statistical finding underscores the significance of each variable as a predictive factor for the log PFU/g recovery from the protein cookie product (Fig. 3).

**Fig. 4:**
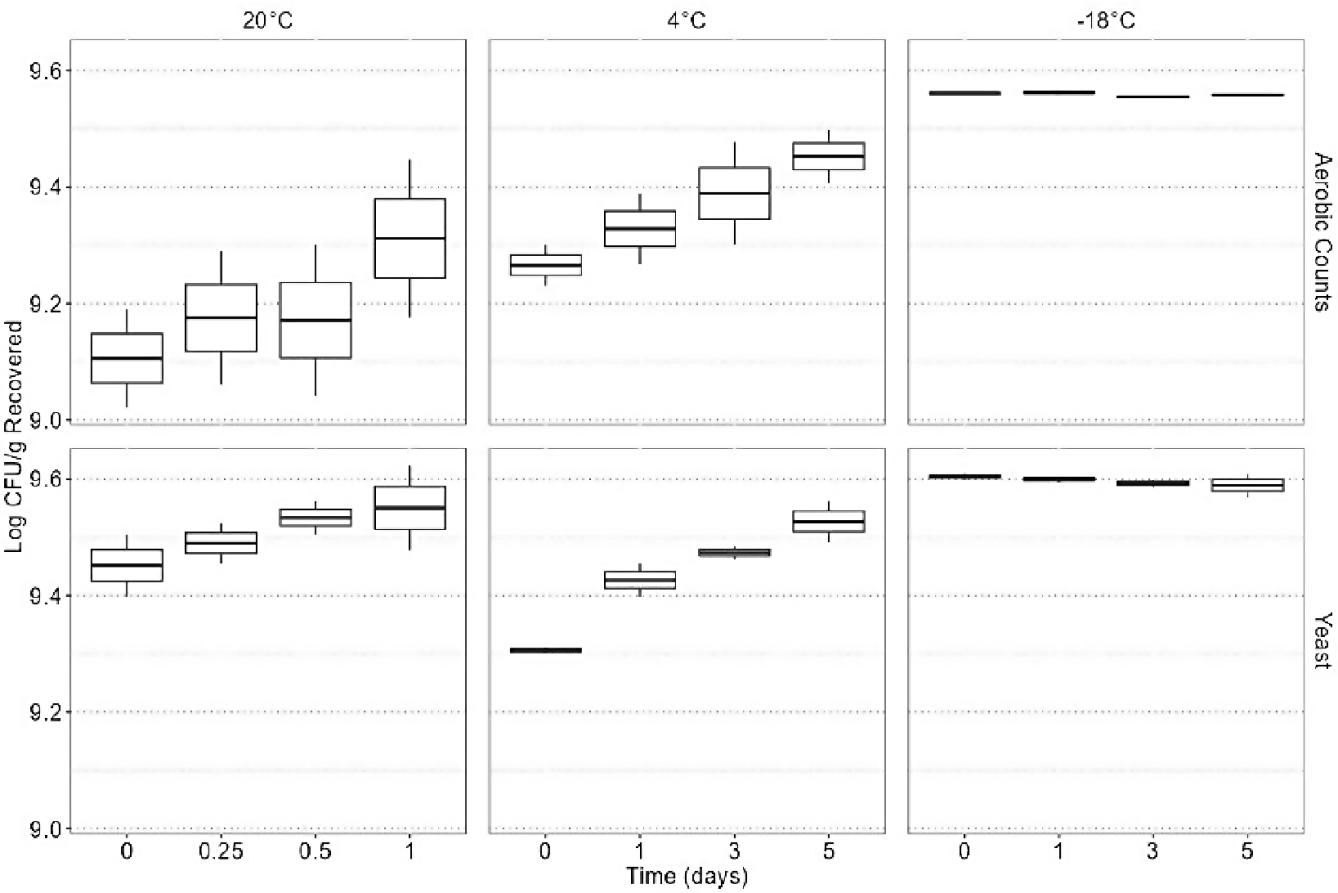
Raw data for quality indicator microorganisms based on time and temperature. In the box plots, the boundary of the box closest to 0 indicates the 25th percentile, the middle line within the box marks the median, and the boundary of the box farthest from zero indicates the 75th percentile. Whiskers above and below the box indicate 1.5 times the interquartile range (the distance between the 25th and 75th percentiles), but are limited to reaching actual data points. Points outside of the boxplots are outliers, that is, values that are outside the range of the whiskers

**Fig. 5:**
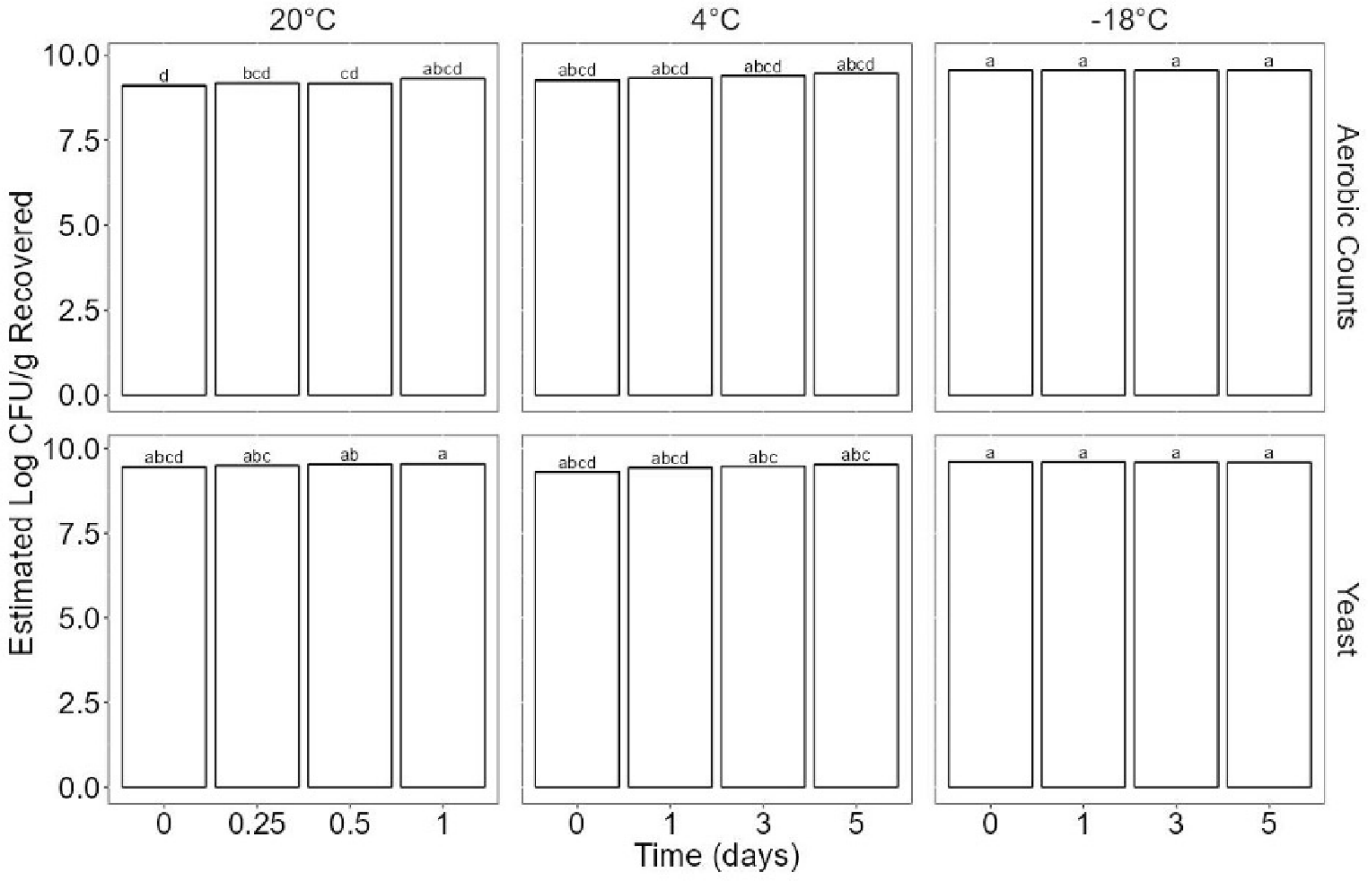
Statistical analysis using ANOVA for quality microorganisms based on time and temperature parameters. Compact letter format is used to designate statistical differences between treatments at *p* = 0.05

All raw data for quality indicator microorganisms are plotted in Fig. 4, which shows the log CFU/g of yeast and aerobic counts recovered from the protein cookie product based on storage time and storage temperature. No molds were detected, thus only yeast counts are reported. A linear model was used to fit the quality indicator microorganism data. A significant two-way interaction effect was found between microorganism type (yeast/aerobic counts) and temperature (*p* = 0.006), indicating that microorganism type and temperature were significant predictors of the log CFU/g recovered from the protein cookie product and time was an insignificant predictor. Yeast and aerobic counts remained steady at all times for each temperature (Fig. 5). Additional enumeration of yeast and aerobic bacteria was performed on the flour and protein powder utilized in the protein cookie food ink and approximately 10.30–10.50 log CFU/g was recovered for both yeast and aerobic bacteria in each material. Notably, the experiments were undertaken in order from highest to lowest temperature over the course of several months, and it appears that starting concentrations of yeast and aerobic bacteria increased in the raw materials as time passed. It is hypothesized that this is the cause of increased counts at time 0 at -18 °C compared to time 0 at 20 °C (Fig. 4).

Mean pH and a_w_ data are given in Table 1. Measured values ranged from 5.80 to 5.87 and 0.8019 to 0.8502 for pH and water activity, respectively. GLMs with Poisson error distributions were used fit to the pH and a_w_ data to determine if storage time or storage temperature were significant predictors of these parameters. Neither model had any significant interaction effects, and both had temperature as a significant predictor with *p* values less than 0.005, in both cases. In short, storage time appears to have no effect on pH or a_w_ under the conditions evaluated here, but storage temperature appears to be a significant factor.

**Table 1:** Mean pH and a_w_ measurements per temperature for initial and final timepoints

Temperature (°C)	Timepoint	pH (mean ± sd)	a_w_ (mean ± sd)
20 °C	0 h	5.83 ± 0.01	0.8386 ± 0.01
	1 d	5.83 ± 0.02	0.8391 ± 0.01
4 °C	0 h	5.86 ± 0.01	0.8237 ± 0.02
	5 d	5.85 ± 0.01	0.8246 ± 0.01
-18 °C	0 h	5.83 ± 0.01	0.8449 ± 0.00
	5 d	5.83 ± 0.00	0.8451 ± 0.00

## Discussion

In this study, fewer TuV virions were recovered in capsules after 24 h at 20 °C. Similarly, at 4 °C, a statistically significant reduction was observed between day 0 and day 1 for both pre- and post- printed storage modalities. No literature regarding persistence of infectious virus particles in 3D printed foods or food inks is known to the authors. Additionally, extant literature predominantly investigates the persistence of enteric viruses during storage vis-à-vis single-ingredient food items. For example, Mormann et al. (2010) documented the absence of discernible reductions in HuNoV titers during refrigeration periods on lettuce (5 days, 11 °C), apples (7 days, 11 °C), and mincemeat (2 days, 6 °C). In a parallel, Lamhoujeb et al. (2008) ascertained a survival period for HuNoV on refrigerated lettuce and turkey greater than ten-days. In-depth investigations, such as those by Baert et al. (2009), have systematically reviewed the effect of refrigerated temperatures on the persistence of diverse enteric viruses across a spectrum of food matrices. Additionally, some research has sought to gauge HuNoV stability through the utilization of surrogate models, albeit with seemingly less data available for TuV. For example, Mattison and coauthors (2007) investigated the survival dynamics of FCV on lettuce and strawberries as proxies for produce contamination via food handlers. A two-log reduction was evident on lettuce subsequent to a 7-day storage period at 4 °C, along with a 2.5 log reduction observed on strawberries following a 6-day storage period at the same temperature (Mattison et al., 2007). Furthermore, Butot and coauthors (2008) conducted an extensive investigation into the survival kinetics of HuNoV surrogates, such as HAV, human rotavirus (RV), and FCV, on cryogenically preserved strawberries, blueberries, raspberries, parsley, and basil. The study concluded that a three-month frozen storage period had minimal effects on HAV and RV infectivity across all tested products, while FCV infectivity exhibited the most substantial decay rate in frozen raspberries and strawberries likely due to acidic pH (Butot et al., 2008). Additionally, Mormann and coauthors (2010) ascertained a lack of statistically significant reductions in HuNoV titer subsequent to the freezing of an inoculated pizza product (7 and 14 days, at − 18 °C) and mincemeat (8 days, − 18 °C).

With regard to pH, available literature documents the stability of TuV to be within the pH range of 3.0 to 8.0, manifesting noticeable reductions at pH 2.5 and 9.0, and achieving greater inactivation at pH 10.0 depending on exposure time (Tian et al., 2013; Arthur & Gibson, 2015). This range for TuV stability aligns with the findings reported in the present investigation, wherein the mean pH was calculated to be 5.84. While a_w_ represents a well-established parameter for comprehending food stability (Rifna et al., 2022), its implications for virus stability remain inadequately characterized (Roos, 2020). Sánchez and Bosch (2016) posit that modifications to the virion surface under heightened relative humidity (RH) levels, coupled with the loss of structural water molecules amid low RH conditions, may contribute to the inactivation of viruses. Lin and Marr (2019) provide additional clarification, asserting that an elevated concentration of solutes at intermediate RH conditions not only mitigates dehydration but also results in an augmented level of virion inactivation. In contrast, Stine et al. (2005) observed that HAV and FCV retained activity under conditions of low RH (45–48%). Moreover, Zhang and coauthors (2021) believe that extremely low a_w_ (a_w_ ≤ 0.25) in conjunction with decreased storage temperature preserved HAV from degradation or reduction in dried produce. In contrast, Trudeau and coauthors (2017) recorded data to support that higher moisture animal feed ingredients had higher survival of Porcine Epidemic Diarrhea Virus (PEDV), Porcine Delta Corona Virus (PDCoV), and Transmissible Gastroenteritis Virus (TGEV). Meanwhile, Lee and coauthors (2015) found no influence of RH on inactivation of HAV, MNV, or MS2 bacteriophage in oysters or peppers. Overall, no consensus has been reached regarding the effects of moisture content/RH/a_w_ on virus persistence in foods.

Prior research suggests that virions exhibit greater resilience within a complex food matrix in comparison to water or simple solutions, as expounded upon by Bertrand et al. (2012) when studying temperature inactivation. Several studies conducted at elevated temperatures (> 50 °C) have proposed a protective effect within certain food matrices, attributed to high levels of protein, fat, or sucrose on the infectivity of foodborne viruses such as HAV and PV (Bidawid et al., 2000; Croci et al., 1999; Deboosere et al., 2004; Murphy et al., 1993; Parry & Mortimer, 1984; Strazynski et al., 2002). It is noteworthy that a restricted number of studies have delved into the stability of HuNoV surrogates within authentic food matrices (Roos, 2020).

In the preparation of food products, assessing microorganisms such as yeast, mold, and aerobic counts as quality indicators is crucial (Sperber, 2007). These microorganisms not only impact the sensory attributes of food but also provide valuable insights into overall quality and shelf life (Duyvesteyn et al., 2001; Fleet, 2011). Sabillón and colleagues (2020) conducted a study on sugar cookie dough, recording yeast and aerobic bacteria at 2.5 ± 0.3 and 3.7 ± 0.1 log CFU/g, respectively. Differing from the protein cookie food ink in the present study, the sugar cookie dough lacked protein powder and included various additional ingredients such as salt, whole egg powder, baking soda, nonfat dry milk, all-purpose vegetable shortening, and vanilla extract. In the prepared food ink of the current study, initial values of 9.45 ± 0.14 and 9.31 ± 0.21 log CFU/g were recorded for yeast and aerobic bacteria, respectively. The discernible differences observed between the two studies are likely attributed to higher concentrations of yeast and aerobic counts (10.30–10.50 log CFU/g) in the flour and protein powder used in the present investigation. These variations emphasize the importance of considering ingredient compositions when interpreting microbial counts in food products.

In light of the present study’s findings, several avenues emerge for prospective research. Foremost among these is the imperative to undertake comprehensive investigations into the interplay among viral persistence, storage conditions, and product characteristics intrinsic to multi-ingredient food matrices. A more expansive analytical approach involving variable a_w_ levels, encompassing a broader spectrum of viral surrogates and product varieties, is warranted to engender a nuanced understanding of these dynamics. Furthermore, elucidating the impact of diverse preservatives or antiviral agents integrated into such food products stands as a valuable trajectory for research, offering insights into strategies for fortifying their safety profiles. Concurrently, in the purview of food safety and quality control, sustained research endeavors should aspire towards the formulation of guidelines and recommendations tailored for manufacturers and regulatory entities. The development of such guidelines could facilitate the secure production and distribution of 3D printed food products. This necessitates a careful consideration of the multifaceted variables pertaining to viral persistence and prevailing environmental conditions, thus contributing substantively to the establishment of a robust framework for upholding the integrity of 3D printed food products within the regulatory landscape.

## Conclusions

The present investigation examined infectious TuV persistence and the dynamics of microbial indicators within the paradigm of 3D printed food, with a specific focus on a protein cookie food ink. Substantive findings included a three-way interaction among time, temperature, and storage method with regard to TuV persistence. Notably, yeast and aerobic counts demonstrated temporal stability, concurrently revealing heightened concentrations within the raw materials. The implications of these findings are significant within the domain of storage protocols and safety considerations, particularly in the context of 3D printed foods. The insights garnered carry implications for regulatory frameworks, offering guidance for the formulation of guidelines for the storage and manipulation of 3D printed foods. Future research directions include investigating the complexities of virus persistence in high-protein foods and exploring various storage conditions and product attributes. In addition, a broader spectrum of viral surrogates and alternative constituents could be tested. Moreover, systematically testing the impact of preservatives, antiviral agents, and other processing methods for enhanced food safety and prolonged shelf life of 3D printed food products is warranted.

## Data Availability

No datasets were generated or analysed during the current study.
